# Outcomes and predictors of respiratory morbidity in children with neuromuscular disorders requiring respiratory support: a cohort study from Vietnam

**DOI:** 10.3389/fped.2026.1911388

**Published:** 2026-08-11

**Authors:** Hung Viet Dau, Tuoi Nguyen Thi, Lam Hoang Kim, Cuong Le Nhat, Canh Ngoc Hoang

**Affiliations:** 1 Pediatric Intensive Care Unit, Vietnam National Children’s Hospital, Hanoi, Vietnam; 2 Department of Pediatrics, University of Medicine and Pharmacy, Vietnam National University, Hanoi, Vietnam; 3 Department of Pediatrics, Hanoi Medical University, Hanoi, Vietnam

**Keywords:** mechanical ventilation, neuromuscular disorders, Pediatric intensive care, prognosis, respiratory failure

## Abstract

**Background:**

Respiratory failure is a major cause of intensive care admission in children with neuromuscular disorders, but data on outcomes and prognostic factors in mixed pediatric neuromuscular cohorts remain limited. This study aimed to describe short-term outcomes and identify predictors of poor outcomes in children with neuromuscular disorders requiring respiratory support in a tertiary pediatric intensive care unit.

**Methods:**

We conducted a single-center cohort study of children aged 1 month to 16 years with neuromuscular disorders requiring respiratory support admitted to a tertiary pediatric intensive care unit in Vietnam between January 2023 and June 2025. Poor outcome was defined as in-hospital mortality, invasive mechanical ventilation for ≥7 days, tracheostomy, or reintubation.

**Results:**

Sixty-three children were included. In-hospital mortality was 14.3% (9/63), and 33 children (52.4%) experienced a poor outcome. Nineteen children (30.2%) required invasive mechanical ventilation for ≥7 days, 10 (15.9%) underwent tracheostomy, and 20 (31.7%) required reintubation. Higher Pediatric Risk of Mortality III (PRISM III) score, shock, lower Glasgow Coma Scale score, and older age were independently associated with poor outcomes.

**Conclusions:**

Respiratory morbidity was substantial among children with neuromuscular disorders requiring respiratory support. Early illness severity and physiological instability at PICU admission were independently associated with poor outcomes.

## Introduction

Neuromuscular disorders (NMDs) in children comprise a heterogeneous group of conditions affecting the anterior horn cell, peripheral nerve, neuromuscular junction, or muscle (1). Although individually uncommon, they represent an important cause of chronic disability and acute respiratory decompensation in pediatric practice. Population-based studies from Europe have shown that pediatric neuromuscular diseases collectively account for a clinically significant burden of morbidity and healthcare utilization (2).

Respiratory compromise is one of the leading reasons for intensive care admission in children with NMDs. Inspiratory muscle weakness, ineffective cough, bulbar dysfunction, secretion retention, aspiration, reduced chest wall compliance, and respiratory infection may precipitate acute respiratory failure requiring escalation of support (3, 4).

Management often requires a spectrum of respiratory therapies, including supplemental oxygen, high-flow nasal cannula, noninvasive ventilation (NIV), invasive mechanical ventilation, cough assistance, and airway clearance strategies. NIV has improved outcomes in selected children with neuromuscular weakness (5), but many still require intubation or prolonged ventilatory support during acute illness (6, 7).

Despite advances in respiratory care and disease-modifying therapies for conditions such as spinal muscular atrophy (8) and Pompe disease (9), critically ill children with NMDs remain vulnerable to prolonged ventilation (9), failed extubation, tracheostomy, and death (10, 11).

Most pediatric literature has focused on single diseases or chronic outpatient management. Data describing mixed neuromuscular cohorts admitted to pediatric intensive care units (PICUs), particularly in low- and middle-income settings, remain limited (12).

As survival of children with neuromuscular disorders continues to improve owing to advances in supportive care and disease-modifying therapies, respiratory complications have become an increasingly important determinant of morbidity, healthcare utilization, and quality of life. Acute respiratory decompensation often represents a critical event in the disease course and may be associated with prolonged ventilatory support, repeated hospitalizations, and functional decline. However, factors associated with poor short-term outcomes during episodes of respiratory failure remain incompletely characterized across heterogeneous pediatric neuromuscular populations. Early risk stratification at PICU admission is clinically important. Identifying children at high risk for deterioration may support timely escalation of monitoring, ventilatory support, airway clearance, and hemodynamic stabilization. However, prognostic factors across heterogeneous pediatric NMD cohorts are not well defined.

We therefore conducted a cohort study of children with neuromuscular disorders requiring respiratory support admitted to a tertiary PICU in Vietnam. Our aims were to describe short-term outcomes and identify predictors of poor outcomes, defined as death or major respiratory morbidity during PICU admission.

## Materials and methods

### Study design and setting

We conducted a retrospective cohort study in the Pediatric Intensive Care Unit (PICU) of a tertiary children's hospital in Vietnam. Consecutive eligible admissions between January 2023 and June 2025 were screened for inclusion using a prospectively maintained clinical database with retrospective verification of medical records.

Our hospital is the largest tertiary pediatric referral center in Vietnam. Children with neuromuscular disorders are managed through a multidisciplinary collaboration involving pediatric intensivists, neurologists, rehabilitation specialists, and respiratory therapists. The hospital provides comprehensive diagnostic evaluation, including genetic and electrophysiological testing, as well as disease-specific therapies when available. Respiratory care resources include high-flow nasal cannula, noninvasive and invasive mechanical ventilation, mechanical insufflation-exsufflation (cough assist), and airway clearance interventions. Pulmonary function testing and sleep studies are available for selected patients outside the acute PICU setting.

### Participants

Children aged 1 month to 16 years were eligible if they had a neuromuscular disorder and required respiratory support during PICU admission. Neuromuscular disorders included conditions affecting the anterior horn cell, peripheral nerve, neuromuscular junction, or muscle, based on the final diagnosis established by the treating neurology and intensive care teams using clinical evaluation and available confirmatory investigations (e.g., genetic, electrophysiological, enzymatic, or imaging studies where applicable).

Respiratory support included one or more of the following: supplemental oxygen therapy, high-flow nasal cannula, noninvasive ventilation, or invasive mechanical ventilation.

Respiratory support was individualized according to the severity of respiratory failure and the patient's underlying neuromuscular condition. HFNC was generally used for children with hypoxemic respiratory failure requiring enhanced oxygen delivery and respiratory support. NIV was considered for patients with preserved airway protective reflexes who exhibited respiratory muscle weakness or hypercapnic respiratory failure but did not require immediate intubation. Invasive mechanical ventilation was initiated in children with severe respiratory failure, inability to protect the airway, progressive respiratory muscle fatigue, or failure of noninvasive respiratory support.

Extubation was considered when patients demonstrated adequate gas exchange on minimal ventilatory support, stable hemodynamics, effective cough and secretion clearance, and sufficient airway protective reflexes. Tracheostomy was considered after multidisciplinary discussion for children with prolonged ventilator dependence, repeated extubation failure, persistent bulbar dysfunction, or an anticipated need for long-term airway support.

Patients with incomplete outcome data were excluded.

### Data collection

Demographic, clinical, laboratory, and treatment variables were extracted from the institutional database and verified against electronic medical records.

Collected variables included baseline characteristics (age, sex, and body weight); diagnostic categories (spinal muscular atrophy, Guillain–Barré syndrome, Pompe disease, muscular dystrophy/myopathy, myasthenic disorders, inflammatory neuromuscular disease, and other neuromuscular diagnoses); admission severity and clinical findings [Glasgow Coma Scale [GCS], Pediatric Risk of Mortality [PRISM III] score, shock, sepsis/healthcare-associated infection, multiorgan dysfunction, bulbar weakness or swallowing impairment, and signs of respiratory distress]; laboratory and respiratory variables [arterial blood gas parameters, including pH, PaCO₂, PaO₂, bicarbonate, and the PaO₂/FiO₂ (PF) ratio, as well as serum creatine kinase and inflammatory markers]; and PICU interventions (noninvasive ventilation, invasive mechanical ventilation, vasoactive support, and tracheostomy). Arterial blood gas analysis was routinely performed at PICU admission for all patients as part of standard clinical care; therefore, PF ratio data were available for the entire study cohort without missing values.

### Definitions

Shock was defined according to the attending intensivist's clinical diagnosis documented in the medical record, supported by persistent signs of inadequate tissue perfusion requiring continuous vasoactive or inotropic support after appropriate fluid resuscitation. Reintubation was defined as reinsertion of an endotracheal tube after planned extubation during the same PICU admission. Prolonged invasive mechanical ventilation was defined *a priori* as invasive ventilation for ≥ 7 days.

### Outcomes

The primary outcome was a composite adverse clinical outcome, defined as in-hospital mortality, prolonged invasive mechanical ventilation (≥7 days), reintubation, or tracheostomy.

Secondary outcomes included in-hospital mortality, PICU length of stay, and duration of invasive mechanical ventilation.

The composite endpoint was selected to capture both mortality and clinically meaningful respiratory morbidity, which may better reflect disease burden in this population than mortality alone.

### Statistical analysis

Continuous variables were assessed for distribution and summarized as median with interquartile range (IQR) or mean with standard deviation, as appropriate. Categorical variables were presented as counts and percentages.

Comparisons between children with and without poor outcomes were performed using the Mann–Whitney U test or Student’s *t* test for continuous variables, and the chi-square test or Fisher’s exact test for categorical variables.

Variables considered clinically relevant or associated with poor outcomes in univariable analyses (*p* < 0.10) were considered for inclusion in the multivariable logistic regression model. Candidate variables included demographic characteristics, underlying neuromuscular diagnosis, admission severity measures, clinical findings, laboratory variables, and respiratory parameters. Multicollinearity among candidate predictors was assessed using variance inflation factors (VIFs), with no evidence of problematic multicollinearity (all VIFs < 2.1). A parsimonious final model was then constructed to minimize overfitting. Adjusted odds ratios (ORs) with 95% confidence intervals (CIs) were reported. All tests were two-sided, and *p* < 0.05 was considered statistically significant.

An exploratory supplementary hierarchical rank-based outcome analysis prioritizing death over major respiratory morbidity was also performed.

Analyses were performed using Stata version 17 (StataCorp, College Station, TX, USA).

## Results

### Study population

During the study period, 63 children with neuromuscular disorders requiring respiratory support were admitted to the PICU and included in the analysis. The cohort comprised children and adolescents with a broad range of neuromuscular diagnoses.

### Primary and secondary outcomes

Among the 63 children, 47 (74.6%) had a previously recognized neuromuscular disorder, whereas 16 (25.4%) presented with new-onset weakness during the index admission. The diagnostic distribution included 17 children with spinal muscular atrophy, 10 with congenital myasthenic syndrome, 9 with Pompe disease, 4 with Duchenne muscular dystrophy, 2 with Guillain–Barré syndrome, 1 with chronic inflammatory demyelinating polyneuropathy, and 4 with weakness of undetermined etiology (Table 1).

**Table 1:** Baseline characteristics according to poor outcome.

Variable	Overall (*n* = 63)	Poor outcome (*n* = 33)	No poor outcome (*n* = 30)	*P* value
Age, years, median (IQR)	4.3 (0.7–9.8)	6.2 (1.7–11.4)	1.6 (0.5–4.6)	0.003
Male sex, *n* (%)	36 (57.1)	20 (60.6)	16 (53.3)	0.560
Neuromuscular diagnosis, *n* (%)				0.584
Spinal muscular atrophy	17 (27.0)	5 (15.2)	12 (40.0)	
Congenital myasthenic syndrome	10 (15.9)	8 (24.2)	2 (6.7)	
Pompe disease	9 (14.3)	3 (9.1)	6 (20.0)	
Duchenne muscular dystrophy	4 (6.3)	2 (6.1)	2 (6.7)	
Guillain–Barré syndrome	2 (3.2)	1 (3.0)	1 (3.3)	
Chronic inflammatory demyelinating polyneuropathy	1 (1.6)	1 (3.0)	0 (0.0)	
Weakness of undetermined etiology	4 (6.3)	2 (6.1)	2 (6.7)	
PRISM III score, median (IQR)	3 (3–7)	6 (3–9)	3 (3–6)	<0.001
Shock on admission, *n* (%)	9 (14.3)	9 (27.3)	0 (0.0)	<0.001
PaCO₂, mmHg, median (IQR)	45 (36–61)	49 (37–71)	41 (35.3–53.3)	0.085
PF ratio, median (IQR)	204 (139.2–317.0)	198.5 (132.9–260.0)	233.3 (183.7–374.4)	0.115

Data are presented as median [interquartile range (IQR)] or *n* (%).

*P* values were calculated using the Mann–Whitney U test for continuous variables and Fisher's exact test for categorical variables, as appropriate.

Overall, 33 children (52.4%) met the composite primary outcome. In-hospital mortality was 14.3% (9/63). The frequencies of the individual components of the composite outcome, including prolonged invasive mechanical ventilation, reintubation, and tracheostomy, are summarized in Table 2.

**Table 2:** Picu course and clinical outcomes.

Variable	Overall (*n* = 63)
PICU stay, days, median (IQR)	7 (3.5–13)
Invasive mechanical ventilation days, median (IQR)	3 (0–8)
In-hospital mortality, *n* (%)	9 (14.3)
Invasive mechanical ventilation ≥7 days, n (%)	19 (30.2)
Tracheostomy, *n* (%)	10 (15.9)
Reintubation, *n* (%)	20 (31.7)
Composite poor outcome, *n* (%)	33 (52.4)

Data are presented as median (IQR) or n (%).

### Comparison between children with and without poor outcomes

Children who experienced poor outcomes were older and had greater illness severity at admission. They had higher PRISM III scores, and shock was observed exclusively in this group (Table 1).

Admission PaCO₂ levels were higher and PF ratios were lower among children with poor outcomes, although these differences did not reach statistical significance. Children with poor outcomes also had longer PICU stay and greater duration of ventilatory support (Tables 1, 2).

### Multivariable analysis

In exploratory multivariable logistic regression analysis, higher PRISM III score, shock, lower Glasgow Coma Scale score, and older age were independently associated with poor outcomes (Table 3).

**Table 3:** Multivariable logistic regression analysis of poor outcome.

Variable	Adjusted OR	95% CI	*p*-value
PRISM III score (per point increase)	1.18	1.03–1.41	**0** **.** **021**
Shock (yes vs. no)	6.42	1.54–28.70	**0**.**011**
Glasgow Coma Scale (per point increase)	0.84	0.72–0.97	**0**.**018**
Age (per 1-year increase)	1.05	1.01–1.10	**0**.**037**

Adjusted ORs are derived from the final multivariable logistic regression model. Variables were selected based on clinical relevance or a univariable *P*-value < 0.10.

Bold values indicate statistically significant associations (*P* < 0.05).

### Sensitivity observations

Although mortality was moderate, the burden of non-fatal respiratory complications was considerable. Most primary outcome events were driven by prolonged invasive ventilation, reintubation, or tracheostomy rather than death alone. This finding supports the clinical relevance of the composite endpoint in this population.

Findings were directionally consistent with the primary analysis, with shock and higher PRISM III scores associated with worse hierarchical ranked outcomes (Supplementary Table 1).

## Discussion

In this cohort of children with neuromuscular disorders requiring respiratory support, in-hospital mortality was moderate, whereas overall respiratory morbidity was substantial. More than half of the patients met the composite poor outcome, largely driven by prolonged invasive ventilation, reintubation, or tracheostomy rather than death alone.

The in-hospital mortality rate of 14.3% observed in our cohort was comparable to previous PICU studies of children with neuromuscular disorders, although direct comparisons should be interpreted cautiously because of differences in patient populations and admission criteria. Nevertheless, mortality alone is likely to underestimate disease burden in pediatric neuromuscular disorders, as many children survive the acute episode but require prolonged respiratory support, repeated airway interventions, or extended PICU stay. Similar observations have been reported in cohorts of children requiring long-term ventilation (13) and complex respiratory care (13, 14).

Although mortality was relatively low, respiratory morbidity remained considerable (15). Nearly one-third of patients required prolonged invasive mechanical ventilation, one-third underwent reintubation, and approximately one in six required tracheostomy. These findings suggest that survival alone does not fully capture disease burden in this population (15–17). Respiratory complications frequently prolonged intensive care and were important contributors to poor clinical outcomes. The composite outcome was designed to capture the overall burden of severe respiratory morbidity rather than mortality alone. Notably, 16 of the 33 children (48.5%) who met the composite endpoint experienced two or more component events, supporting the use of this composite measure to reflect clinically meaningful respiratory deterioration rather than isolated adverse events.

Our findings also support the clinical value of composite outcomes that incorporate both survival and clinically meaningful respiratory morbidity, particularly in specialized centers where mortality is relatively uncommon but resource-intensive respiratory complications remain frequent. Notably, most adverse events in our cohort were related to respiratory morbidity rather than death. Prolonged invasive ventilation, reintubation, and tracheostomy are clinically important outcomes because they are associated with increased healthcare utilization, longer hospital stay, caregiver burden, and potential long-term respiratory dependency. These outcomes may therefore be particularly relevant when evaluating the effectiveness of acute management strategies in children with neuromuscular disorders.

Shock, higher PRISM III score, lower Glasgow Coma Scale score, and older age were independently associated with poor outcomes. This is biologically plausible, as children with neuromuscular weakness may have limited physiological reserve and may deteriorate rapidly when faced with sepsis (18), pneumonia, dehydration, cardiopulmonary dysfunction (19), or multiorgan failure (18, 19).

PRISM III has been widely used as a pediatric severity score and remains associated with mortality risk across mixed PICU populations (20). Our results suggest that global physiological derangement at admission may provide stronger short-term prognostic information than diagnosis alone. An important consideration is that the clinical landscape of pediatric neuromuscular disorders is changing rapidly with the introduction of disease-modifying therapies for conditions such as spinal muscular atrophy and Pompe disease. Although these therapies have improved survival and motor outcomes, respiratory complications remain a major cause of hospitalization and intensive care utilization. Consequently, the identification of predictors of acute respiratory deterioration remains clinically relevant even in the modern treatment era. Our findings suggest that acute physiological instability at presentation may continue to influence short-term outcomes regardless of underlying diagnosis.

Older age was independently associated with poor outcomes in our cohort. Although older children may have accumulated greater disease burden and more advanced respiratory muscle weakness, this interpretation remains speculative. Alternative explanations include differences in the distribution of underlying neuromuscular disorders, referral bias inherent to a tertiary referral center, variations in treatment strategies, and a higher prevalence of chronic respiratory complications among older children. Because these factors were not systematically evaluated in our study, the mechanisms underlying this association remain uncertain and warrant further investigation. Accordingly, respiratory morbidity may be an increasingly relevant outcome measure alongside mortality in contemporary neuromuscular populations.

Children with poor outcomes also showed a trend toward higher PaCO₂ levels and lower oxygenation indices at admission. Although these differences were not statistically significant, they are physiologically plausible and may reflect greater ventilatory muscle weakness, hypoventilation, secretion burden, or concurrent pulmonary infection. These findings should be interpreted cautiously given the modest sample size.

Although etiologic diagnosis remains essential for disease-specific treatment, diagnostic subtype alone showed less prognostic value than bedside severity markers. Despite the heterogeneous diagnostic spectrum represented in our cohort, admission severity markers consistently demonstrated greater prognostic value than the underlying neuromuscular diagnosis. Children with very different underlying disorders may converge clinically through the same final pathway of respiratory failure, secretion burden, and circulatory instability.

This observation is relevant in settings where confirmatory testing may be delayed. Early clinical assessment of shock, consciousness level, gas exchange, and need for escalating respiratory support may be more immediately actionable than diagnostic labeling. From a clinical perspective, recognition of shock, impaired consciousness, and greater illness severity at PICU admission may facilitate earlier escalation of monitoring, respiratory support, airway clearance interventions, and hemodynamic stabilization. These readily available bedside variables may be particularly useful in resource-limited settings where access to advanced diagnostic testing is delayed or restricted.

Children with neuromuscular disorders are particularly vulnerable to respiratory deterioration because of inspiratory muscle weakness, ineffective cough, bulbar dysfunction, scoliosis, chest wall restriction, and impaired airway clearance. These mechanisms promote secretion retention, atelectasis, recurrent infection, extubation failure, and prolonged ventilatory support. Previous respiratory care guidelines therefore emphasize airway clearance techniques (4), non-invasive ventilation (21), cough augmentation (22) and secretion management as key components of management.

Previous studies of critically ill children with neuromuscular disorders have largely focused on specific diseases or single-center cohorts. Despite advances in respiratory care and disease-modifying therapies, recent reports continue to demonstrate substantial respiratory morbidity (12) and ongoing dependence on intensive respiratory support among affected children (23).

Our study extends these observations to a contemporary cohort from Vietnam and highlights the prognostic importance of acute severity markers in a mixed-diagnosis population. Data describing critically ill children with neuromuscular disorders remain limited in low- and middle-income countries. Our findings contribute contemporary evidence from Southeast Asia and may help inform risk stratification in settings with similar healthcare resources. The heterogeneous nature of the cohort may also be viewed as a strength. Although the included disorders differ in etiology, many converge clinically through common pathways of respiratory muscle weakness, ineffective cough, secretion retention, and respiratory failure. Consequently, a mixed-diagnosis cohort may better reflect the spectrum of neuromuscular disease encountered in routine pediatric intensive care practice than disease-specific studies alone. The identification of shared prognostic factors across different neuromuscular disorders may therefore have broader clinical applicability.

This study has several limitations. It was conducted at a single tertiary center with a relatively small sample size, which may limit generalizability and the stability of the multivariable model despite restricting the number of predictors. Consequently, the identified predictors should be interpreted cautiously and validated in larger multicenter cohorts. Some variables were derived from routinely collected clinical data and may have been incomplete, including information on pre-PICU respiratory support. Long-term functional outcomes after discharge were unavailable. Finally, secondary analyses of individual components of the composite endpoint were exploratory and should be interpreted descriptively, although the hierarchical rank-based sensitivity analysis yielded consistent findings.

## Conclusions

Among children with neuromuscular disorders requiring respiratory support in the PICU, mortality was moderate but respiratory morbidity was substantial. More than half experienced death or major respiratory complications. Shock at admission, greater illness severity, lower Glasgow Coma Scale score, and older age were independently associated with poor outcomes.

## Data Availability

The original contributions presented in the study are included in the article/Supplementary Material, further inquiries can be directed to the corresponding author.
