# Supplementary material for: Outcomes and predictors of respiratory morbidity in children with neuromuscular disorders requiring respiratory support: a cohort study from Vietnam
Source: Front Pediatr. 2026 Aug 11;14:1911388. doi: 10.3389/fped.2026.1911388 (PMC13503341; doi:10.3389/fped.2026.1911388)
Supplement: Supplementary file 1 [file Table1.docx]

**Supplementary Table S1. Exploratory hierarchical rank-based outcome analysis**

| **Predictor** | **Statistical test** | **Effect direction** | **P value** |
| --- | --- | --- | --- |
| Shock vs no shock | Mann–Whitney U | Worse outcomes with shock | **0.001** |
| PRISM III score | Spearman correlation | Higher score = worse outcomes | **0.034** |
| Age | Spearman correlation | No clear association | 0.732 |
| **Footnote:** Ranked outcome prioritized death, tracheostomy, prolonged invasive ventilation (≥7 days), reintubation, and no major event. | | | |
